# Factors affecting aseptic loosening in primary total knee replacements: an in vitro study

**DOI:** 10.1186/s40634-020-00243-9

**Published:** 2020-06-05

**Authors:** David Lionberger, Laura Wattenbarger, Christopher Conlon, Timothy J. Walker

**Affiliations:** 1 Southwest Orthopedic Group, The Methodist Hospital at Houston, 6560 Fannin Street, Suite 1016, Scurlock Tower, Houston, TX 77030 USA; 2 Texas A&M College of Medicine, The Methodist Hospital at Houston, 6565 Fannin, Street, West Pavilion 5, Houston, TX 77030 USA; 3 The University of Texas Health Science Center at Houston, 7000 Fannin Street #1200, Houston, TX 77030 USA

**Keywords:** Primary total knee, Cement, Adhesion, Bonding, Viscosity, Application time, Coat implant, Surface

## Abstract

**Background:**

Implant surface integrity and cement bonding are assumed to be sufficient in primary total knee replacements to stabilize implants for extended wear without concerns over delamination and loosening. Yet there exists a significant rate of aseptic loosening where failure at implant cement interface occurs. The aim of this study is to look at specific aspects leading to aseptic loosening of the total knee replacement, where cement adhesion to the implant results in the lowest pull off strength.

**Methods:**

Virgin ceramic coated and uncoated chrome cobalt tibial trays were used in a pull off study using differing viscosities of cement at varied time intervals to compare which combination is strongest compared to which is least resistant to pull off testing.

**Results:**

Low viscosity cement had a 44% (5.9 kg verses 3.3 kg, *p* < 0.001) higher pull-off strength compared to high viscosity cement. Coated implants had a 30% (3.9 kg verses 5.5 kg, *p* = 0.037) lower pull-off strength compared to non-coated. Testing measures were limited to cement utilization less than 5 minutes due to the poor adhesion of the dowels beyond this time. Finally, there was a significant difference in adhesion properties between brand names when utilizing low viscosity cement on the non-coated trays (10.34 kg for Simplex verses 4.87 for Palacos, *p* = 0.021).

**Conclusion:**

There are differences in adhesion properties between cement vendors, prompting significant concerns over the use of coated implants with particular cement types. Use of low viscosity cement on non-coated surfaces in the early liquid phase of cement curing was found to produce the best chance for adequate adhesion. This study demonstrates that there is variation in the adhesive properties of implants utilized in total knee replacements, and that the orthopedic community should consider not only the implant, cement, and curing time individually, but the overall integrity conferred from the combination of all of these variables.

## Background

Worldwide registry data show failure of total knee replacements on the order of less than 1% per year [17–19]. Aseptic loosening in most series makes up the bulk of failures, ranging from 22.8 to 31.2% [3, 23]. Much has been written about balance, alignment, size disparity and rotational aspects to help replicate the native anatomy and help limit these failures [1, 5, 10, 15, 25]. At the core of the implant longevity is the durability and strength of the cement on the metal interface.

Since the advent of cement in the mid 1940s, there remains a paucity of recent literature on cement adhesion strength. The use of cementless total knee replacements has not occurred as rapidly as it has in total hip replacements [3, 6, 11, 27]. Since cement remains integral to secure component fixation, it is important to not have a cavalier view of its capabilities and limitations [16, 21, 23]. A series of failures occurred in a cohort of coated implants using the same traditional cement technique and conditions that had been used for the proceeding 30 years [17–19]. We found a 3% gross loss and the 6% at-risk of failure, noted by progressive cement loosening, implants over a 6-month period, rather than the expected 0.5% failure per year rate seen in the past.

**Fig. 1:**
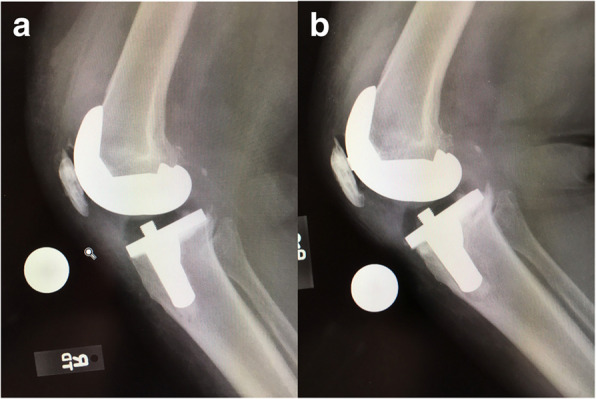
The classic loosening of the implant in worst instances begins with a delamination pull-away of the cement on the posterior keel, followed by failure of the proximal posterior tibial surfaces; Compare the positioning immediately post-op (**a**) verses that at 6 months, where the slope changes from 7^o^ to 9^o^ and the implant subsides (**b**)

The first finding notable in these failed cases was a systematic chronological loosening of the posterior keel at 6 to 12 months, followed by an almost reproducible failure of the entire surface of the tibia (Fig. 1). After revising two of these implants, it was suspected that there might be a failure of bonding to the surface of the implant itself, causing the sequence of events to occur. This sequence of failure (keel, tibia, then femoral component of anterior flange) was later to be reproduced in a cohort of 8% of the group of implants showing debonding in an average follow up of 1.5 years compared to an expected 0.7% per year [19].

It was felt compression or sheer failure was not the issue, which much of cement research focuses on, but instead adhesion failure. While establishing a power analysis, if the implant cement was not applied to the surface of the implant within 5 min, which is well under the stated manufacturer’s “application” or “workable phase,” cement adhesion was inadequate to assemble the testing apparatus. Early failure occurring only in the coated implants in the sequential series of patients where this coated implant was used. With this in mind, we set about to test both the installation of cement with respect to time on two different surfaces.

The aim of this study is to establish the best scenario of surface type with respect to coated status, cement type and viscosity and time to application by a selective variance of all three variables. We hypothesize that surface coating, delay of time, and high viscosity may adversely change bonding strength.

## Methods

Two types of surfaces were tested utilizing unused, virgin implants to determine pull-off strength comparisons. One surface was the VEGA Aesculap (Tuttlingen, Germany) with zirconium advanced surface (AS) coating on the back of tibial implants. The second surface was the non-coated cobalt chrome version of the same tibial tray, which had undergone an identical grit blasting and surface roughening preparation for cement application. It is notable that the surface roughness is identical in both implants in that the coating applied to the AS variety does not change or dampen the roughness characteristics. A total of three cements were used in testing: Simplex low viscosity (SLV) manufactured by Stryker (Mahwah, NJ), Palacos low viscosity (PLV) and Palacos high viscosity (PHV) manufactured by Heraeus Medical (Vardley, PA). The differing viscosities allowed for the measurement of a potential confounding variable.

**Fig. 2:**
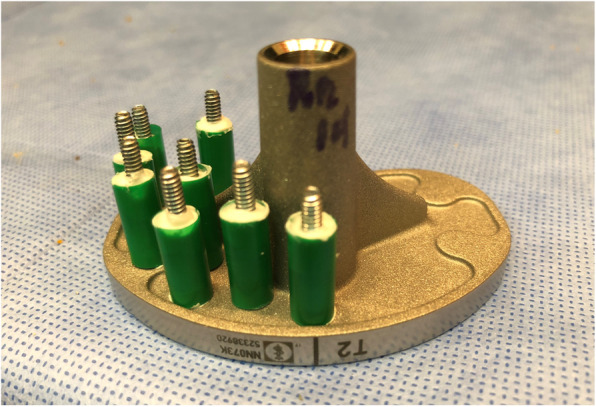
Tibial tray showing the first phase of dowel application, as many as five cement mixes may be utilized to complete a fully utilized tray area

The testing samples utilized a 4.5 mm straw tube mold cut at 1 cm in length, which was injected with the test cement. A pull-off screw with head embedded in the test cement was utilized to reproduce consistent pressures exerted on the pull-off dowel in extraction testing, as seen in Fig. 2. Cement mixing was at a constant 20 degrees Celsius temperature at 50% humidity, and the strength testing began at 30 s and continued after mixing until 5 min of post-mixing time in all series. To prepare testing cement specimens for each trial, 5 dowels were prepared at one-minute intervals, spanning from 1 minute to 5 minutes after the cement was mixed. If the dowel was not placed within 15 s of its assigned interval, it was moved to a subsequent time interval. This rather abbreviated application time range was strictly adhered to during testing to encompass the best “workable phase” wisdom defined by vendors, as we found during the pre-testing that a delay in application of cement beyond 5 min created such a poor adhesion that it eliminated any useful surface bonding and as a result would skew data to such a low adhesion level that comparison differences would not be apparent. As a note, this “workable phase” is derived from Federal guidelines of cement performance standards from International Organization for Standardization Documents (ISO) as well as the American Society for Testing and Materials (ASTM) [2, 13].

With respect to the testing protocol, the in vitro samples were incubated in normal saline for 2 weeks before the pull-off studies were performed. The pull-off studies were recorded in real-time, so as to determine the maximum tension applied pull-off strength before failure. Each pull-off dowel was tested to failure without toggle or torsional twisting in a suspended, direct pull linear fashion using a digital tensometer with sensitivity to 0.001 kg and camera recordings. This allowed for the time of failure, at which the maximum force applied caused the dowel to detach from the test tray, to be accurately recorded. Data were then assembled using time analysis for the type of cement, viscosity subtype, and surface utilized. It was then stratified based on each cement-surface combination, with 153 data points in total, 76 uncoated and 77 coated tray samples of which the various viscosities were equally represented (Table 1).

**Table 1:** Distribution of testing samples utilizing different cement brands of both high and low viscosity's tested on coated and non-coated tibial trays

	Average	Stdev	CI	N
**NC PHV**	2.47	1.94	0.67	32
**C PHV**	4.25	3.95	1.39	31
**NC PLV**	4.87	3.44	1.14	35
**C PLV**	3.35	1.91	1.88	4
**NC SLV**	10.34	5.55	2.22	24
**C SLV**	3.67	3.66	1.38	27
**Refobacin Poly**	0.64	0.58	0.38	9
**Palacios Poly**	0.64	0.61	0.49	6

*NC* non-coated, *C* coated, *PHV* palacos high viscosity, *PLV* palacos low viscosity, *SLV* simplex low viscosity

Total sample size testing of 153 implant cement combinations were analyzed. A series of linear regression models were used to compare the pull-off strength for each comparative group. The first model included the viscosity of the cement as the independent variable, the second model included coating status as the independent variable, and the final model included both viscosity and coating status with an interaction term between the two variables. An additional linear regression model examined the association between time and pull-off strength. A *p*-value of < 0.05 was used to indicate statistical significance. The analysis was conducted using Stata 15.0.

## Results

With respect to cement viscosity, the cement bonding strength was significantly lower (*p* < 0.001) in high viscosity cement (PHV) when compare to low viscosity cement (SLV and PLV). Averaged pull off strength for SLV and PLV together was 5.9 kg, versus that of high viscosity at 3.3 kg, implicating cement bonding strength is reduced by 44% when utilizing high viscosity cement over low viscosity cement. When individually considered, PLV had a bonding strength of 4.7 kg and SLV had a bonding strength of 6.8 kg, both of which were statistically higher than that of high viscosity cement. Additionally, SLV was found to have a statistically higher cement bonding strength than PLV (*P* = 0.021 with 95% CI of 0.319 to 3.871). The second variable examined was coating status. There was a statistically significant reduction (*P* = 0.037 with 95% CI of − 2.963 to − 0.096) in cement bonding strength in the AS coated implants when compared to non-coated implants. AS coating pull off strength was 3.9 kg versus that of the non-coated implants at 5.5 kg. This is a 30% overall reduction in pull off strength.

**Fig. 3:**
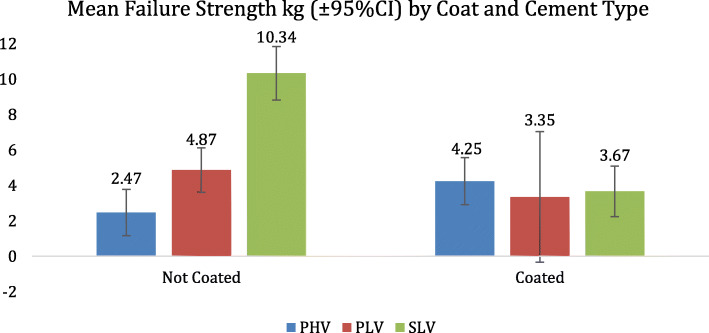
Graph showing the variation in pull-off strength based on combinations of cement type and coating status

Based on these findings, we directed our analysis towards a combination of the two variables to determine the most and least optimal combination. The graph (Fig. 3) displays the relative strength seen in coated implants compared to that of uncoated implants relative to various brands and viscosities of cement. Non-coated SLV was found to have the highest average pull-off strength of 10.34 kg, whereas non-coated-PHV was found to have the lowest average pull-off strength of 2.47 kg (*P* < 0.05 with 95% CI of 5.875 to 9.865). PHV used with non-coated implants was found to show the lowest bonding strength, at 26% of the strength, compared to the greatest bonding strength of SLV used with non-coated implants.

The longer the elapsed time following mixing, the lower the pull-off strength attained. The overall trend of decreasing pull-off strength as the time to application increased suggests inferior implant-surface bonding as the cement was able to cure before application. However, there was no statistically significant decrease in pull-off strength for all implants with respect to time elapsed (*P* = 0.298 with 95% CI of − 0.781 to 0.241).

## Discussion

The most important findings that our study established include a reduction in bonding strength in high viscosity cement verses low viscosity cement (*P* < 0.001), an overall trend between time to application and pull-off strength, and a reduction in bonding strength on coated verses uncoated surfaces (*P* = 0.037). Although multifactorial failures of this variety are often difficult to predict and even more difficult to ascertain cement adhesion deficiencies, it appears that at least two, if not three, combinations may be in play for suboptimal fixation. Clearly the cement viscosity link to the surface technology is vital.

Secondly, this study suggests there to be an overall inferior fixation of cement to a coated surface. What is interesting, however, is the individual trend for particular cement types with respect to coating status. The finding that each cement type responds differently to coating status, as seen in Fig. 3, that implores a further exploration of the current implant-cement combinations being utilized.

Finally, this study shows that there is an unrecognized variation in implant adhesive properties. Our conclusion that low viscosity cement is statistically superior to high viscosity cement in regards to pull-off strength is consistent with prior findings published in the previous few years of high viscosity cement having a higher susceptibility for aseptic loosening of the tibial component than other cements [3, 6, 11, 12, 14]. Ultimately, coated implants used with high viscosity cement may have inferior adhesion strength, which could be detrimental to implant durability. Despite this, the integrity of the coated surface has been suspect for some time. Fixation of cement is not a covalent atom-to-atom link, but instead a dipole-dipole interaction that creates polarization effects on a mechanical roughened substrate. As this is exposed to water, bonding strength decreases, resulting in lower yield strength from hydrolyzation [7]. Secondly, crack propagation in cement may also weaken the cement at the bond junction. Pre-treatment with SiOx has been reported to greatly enhance bond strength, creating Van der Waals ionic bonds with the silicon monoxide layer, stabilizing the hydrolytic debonding while lessening surface crack propagation [20, 22]. However, the pre-treatment is not present on this implant or others to our knowledge.

### Strengths and limitations

Weakness and limits exist in any study, as ours is not immune in this particular scenario. The most profound is the extremely narrow “workable phase” where extended time caused massive weakness in cement strength. In this series, samples were never tested beyond 5 minutes to lesson confounding variables of cement adhesion. Also, a number of dowel plugs extruded cement despite attempts to clean around the base, which may affect adhesion, as the surface area would be larger than the 4.5 mm cone. We sought to limit this by making an area normalization adjustment. Extruded cement represents a confounding variable inherent in any type of force study, of which this study is no exception.

We did not test any other makers of implants, but merely looked at the surface coating as a standard amongst the brand name utilized in this case. While this does not condemn surface technology modifications, it by no means excuses the importance at looking at what that may do in terms of the fixation long-term. There have been some questions regarding adhesion durability of cement fixation in vivo. The longer the implant remained in the patient, the more likely cement would loosen on tibial trays [8, 24]. This was confirmed in vitro studies, where after 60 days of incubation, strength of cement adhesion on ceramic coated cobalt chrome was completely unbonded [4, 26]. Comparatively, given similar surface there is no published data on differences in chrome cobalt and titanium. It is of note that the surface roughness of the coated implant is identical to the chrome cobalt. One report of pull out strength noted similar strength. However, a trend for inferior cement adhesion to ceramic was noted in the published data. This study was flawed due to the complexity of failure modes of sheer and bonding pull off failure that were not isolated [9]. It also should raise some concern in the orthopedic community that there are in fact differences in adhesion properties between certain cement brands and may extend to the technique the surgeon utilizes in terms of early verses late curing phase. The high viscosity cement is by far easier to work with than the low viscosity; however, if one is compelled to use coating systems, it may well serve the surgeon to ensure these surfaces have been tested to failure and not merely tested on what federal guidelines, ASTM, and ISO standards suggest are appropriate. It is also apparent that while we did not test cement in the late phase of curing but instead in the early “sticky” phase, one can only imagine what the results of our testing, let alone the clinically implications, would have been using cement during the late phases of curing. It is also important that our study, as are the majority of those cited above, is an in vitro study.

Additional sources of potential limitations include the size of the data set and the distribution of the data set. The size was relatively small, with a total of 153 implant-cement combinations tested. Ideally, the findings from our data would prompt a more extensive and larger exploration of the limitations of current cement, which would reduce any confounding variables that might be present in our data set. We found that our data were positively skewed (skewness = 1.7); however, this is unlikely to negate our findings and would be limited as well by future testing with more data points.

## Conclusion

What is more important to us in the orthopedic community is to constantly be exercising diligence to test beyond mere standards established by the U.S. Government to satisfy certain pre-existing testing criteria that the guidelines would suggest. In this case, these guidelines were insufficient. When one is establishing a superior surface, one also looks to that surface as being a disruption for a bonding surface, which should raise red flags with those of us who use these and place our trust in them. Until we develop a more astute testing standard, we should implore ourselves to not be lulled into thinking that performance of the implant is immune to mandates for superior fixation capabilities.

This study points out the importance of cement, surface, and design in assuring the best results for our patients, less we as orthopedic surgeons become complacent in the more common aspects of our practice. This information is hopefully not the end of the research that will be forthcoming in the years to come. While this information did identify the perfect storm for failure in these particular implants, it should be a lesson to all of us that technology can be helpful on the one hand but detrimental in another. It seems a logical conclusion that a superior wear surface would be harder to bond to, yet in previous years, it did not strike either the senior surgeon or the vendors promoting these superior technologies as something to be considered. This paper should serve as a springboard for future discussion regarding this disquieting set of findings.

## Data Availability

The datasets used and/or analyzed during the current study are available from the corresponding author on reasonable request.
